# A Computed Tomographic Study of the Premolar Teeth of *Babyrousa* spp.

**DOI:** 10.1177/08987564231166551

**Published:** 2023-03-30

**Authors:** Alastair A. Macdonald, Bianca Ziehmer, Andrew C. Kitchener, Magnus Gelang, Björn Åblad, Ruth Lintonsson, Kerstin von Pückler, Sebastian Schaub, Ingmar Kiefer, Tobias Schwarz

**Affiliations:** 1 Royal (Dick) School of Veterinary Studies & The Roslin Institute, University of Edinburgh, Edinburgh, UK; 2 Department of Viticulture and Agriculture, Ministry of Economic Affairs, Transport, Agriculture and Viticulture Rhineland Palatinate (MWVLW), Mainz, Germany; 3 Department of Natural Sciences, 48013 National Museums Scotland, Edinburgh, UK; 4 Institute of Geography, School of Geosciences, University of Edinburgh, Edinburgh, UK; 5 Göteborgs Naturhistoriska Museum, Goteborg, Sweden; 6 Blåstjärnans Djursjukhus, Bildenheten, Göteborg, Sweden; 7 Justus-Liebig-Universität Giessen, Klinik für Kleintiere, Radiologie, Justus-Liebig Universität, Gießen, Germany; 8 Universität Leipzig, Veterinärmedizinische Fakultät, Klinik für Kleintiere, Leipzig, Germany

**Keywords:** anatomy, wild pig, babirusa, *Babyrousa* spp., tooth root, root canal

## Abstract

A photographic and computed tomography (CT) scanning study was carried out on the premolar teeth of 18 adult male *Babyrousa babyrussa* skulls, 10 skulls of *Babyrousa celebensis*, including 6 adult males, 1 adult female, 1 subadult male, 1 subadult female, and 1 juvenile male. The occlusal morphology of the permanent maxillary premolar teeth of *B. babyrussa* was very similar to that of *B. celebensis*. Almost all the maxillary third premolar teeth (107/207) had 2 roots, whereas maxillary fourth premolar teeth (108/208) had 3 or 4 roots. All of the mesial tooth roots of 107/207 and 108/208 were tapering rod-like structures; each contained a single pulp canal. Almost all distal roots of 107/207 were “C” shaped and contained 2 pulp canals. The 108/208 palatal roots were “C” shaped and contained 2 pulp canals. The mesial and distal roots of the mandibular third premolar teeth (307/407) teeth were uniformly rod-like, as were the mesial roots of the mandibular fourth premolar teeth (308/408) teeth. The distal roots of the 308/408 teeth were “C” shaped. All *B. babyrussa* 307/407 teeth have a single pulp canal located in each of the mesial and distal roots. The 308/408 mesial tooth root contained 1 pulp canal. In all but 3 of the 36 distal 308/408 roots of *B. babyrussa* teeth and in 7 of the 14 distal roots of *B. celebensis* teeth there was a single pulp canal; in the other 7 teeth there were 2 pulp canals. Each of the 3 medial roots contained 1 pulp canal.

## Introduction

The babirusa (genus *Babyrousa*)^1^ is a suid endemic to eastern Indonesia, inhabiting the islands of Buru; the Sula Islands of Sehu, Taliabu, and Mangole; the island of Sulawesi; and the Togian Islands.^2–4^ Early anatomical investigations of the appearance of the teeth in the adult male babirusa, based on the skulls of animals from Buru, have been reported in the literature.^5–9^ The male permanent maxillary dentition comprises 4 incisor, 2 canine, 4 premolar, and 6 molar teeth. The corresponding mandibular dentition contains 6 incisor, 2 canine, 4 premolar and 6 molar teeth. The female may, or may not, have canine teeth.^6–9^ Illustrations of the teeth^9,10^ raised the hypothesis that the roots underlying the premolar teeth were not simple in form.

**Figure 1.**
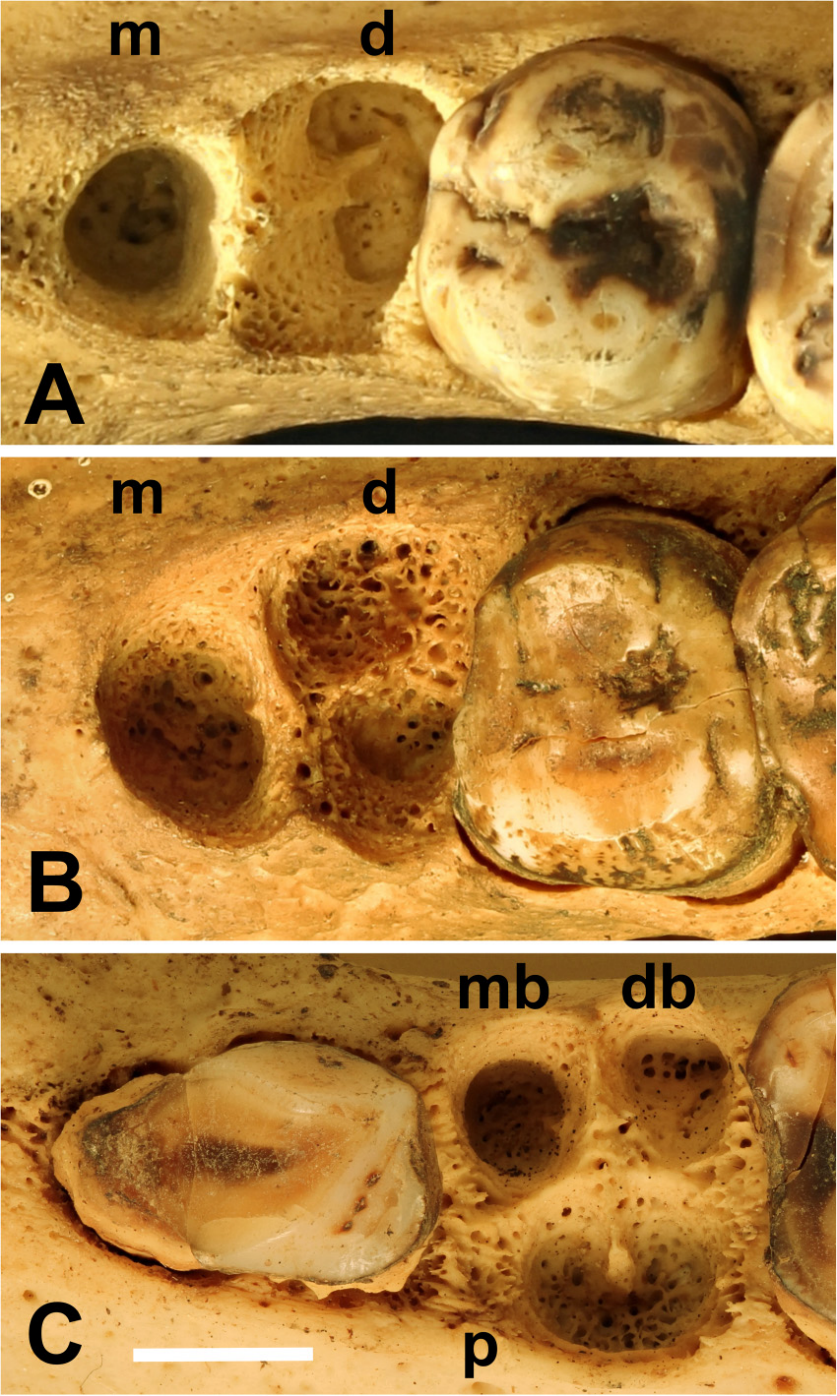
*Babyrousa celebensis* skulls showing maxillary premolar tooth alveoli. (A) Right maxillary third premolar (107) alveolus (NMS,Z,1930.172). (B) 107 alveolus (GNM 17.938). (C) Left maxillary fourth premolar (208) alveolus (GNM 17.941) illustrating differences in maxillary premolar tooth root number and shape. Abbreviations: d, distal; db, distobuccal; m, mesial; mb, mesiobuccal; p,  palatal (bar = 5 mm).

In the course of recent studies of babirusa skulls,^11–14^ a number of maxillary and mandibular specimens were found to have missing teeth. These observations added to the suggestion that among the babirusa there were variations in the number, sizes, and shapes of the premolar tooth roots. Approximately round-shaped and oval-shaped tunnels were observed running into the spongy (cancellous) bone of the maxilla and mandible (Figure 1); the cross-sectional shape of some of these alveoli^15^ were similar to the letter “C” with a bony core to the “C” (Figure 1A and C). This shape and a review of its development have been described.^15^

A search of the literature failed to reveal the results of any systematic study of the anatomy of the premolar teeth of babirusa. And yet, clinical veterinary dentistry has begun to be undertaken on the babirusa.^16,17^ As a consequence, we undertook a computed tomography (CT) examination of babirusa skulls from 4 museum collections^a–d^. The skulls selected represented the babirusa species from Buru and the Sula Islands (*Babyrousa babyrussa*) and from the island of Sulawesi (*Babyrousa celebensis*).

**Figure 2.**
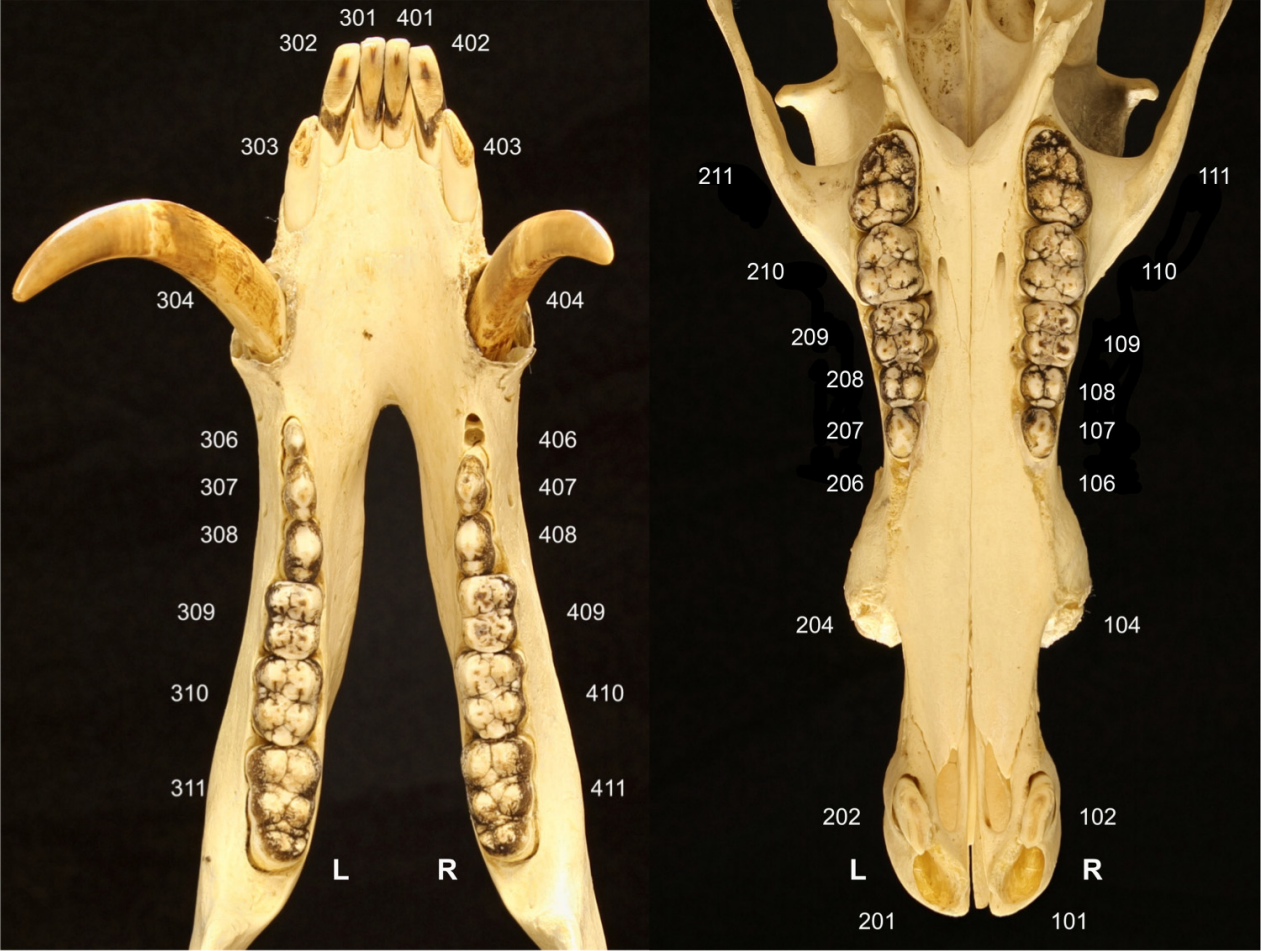
Illustration of the modified triadan system of tooth numbering applied to the babirusa (*Babyrousa babyrussa*) (NMS,Z,2002.210.1).

The modified triadan system of veterinary tooth nomenclature was adopted for this study^18^ and is illustrated in Figure 2. The veterinary dental nomenclature^19^ was used to provide the description of dental location and orientation. The positions of persistent deciduous teeth, when present, were allocated the corresponding identification numbers 106 and 206 on the maxilla and 306 and 406 on the mandible.

## Materials and Methods

The research material comprised 13 adult male *B. babyrussa* skulls from Buru, 5 adult male *B. babyrussa* skulls from Buru or the Sula Islands and 6 adult male and 1 adult female *B. celebensis* skulls from Sulawesi (Table 1). An additional 3 *B. celebensis* skulls from Sulawesi were studied; 1 juvenile male, 1 subadult male, and 1 subadult female.

**Table 1.** *Babyrousa babyrussa* and *Babyrousa celebensis* Skulls Studied.

Specimen ID	Museum	Museum catalogue number	Geographical origin	Sex	Age
AAM0211	DresdenMuseum	607	Buru	M	Adult
AAM0212	DresdenMuseum	608	Buru	M	Adult
AAM0214	DresdenMuseum	1554	Buru	M	Adult
AAM0215	DresdenMuseum	555	Buru	M	Adult
AAM0216	DresdenMuseum	1556	Buru	M	Adult
AAM0218	DresdenMuseum	2165	Buru_or_Sula	M	Adult
AAM0220	DresdenMuseum	2167	Buru	M	Adult
AAM0237	Edinburgh-NationalMuseumsScotland	NMS.Z.1991.15.1	Buru_or_Sula	M	Adult
AAM0239	Edinburgh-NationalMuseumsScotland	NMS.Z.1991.15.3	Buru_or_Sula	M	Adult
AAM0240	Edinburgh-NationalMuseumsScotland	NMS.Z.1991.15.5	Buru_or_Sula	M	Adult
AAM0243	Edinburgh-NationalMuseumsScotland	NMS.Z.1992.10.28	Buru	M	Adult
AAM0257	Edinburgh-NationalMuseumsScotland	NMS.Z.2001.142	Buru	M	Adult
AAM0260	Edinburgh-NationalMuseumsScotland	NMS.Z.2002.210.1	Buru	M	Adult
AAM0287	FrankfurtSenckenbergNaturalHistoryMuseum	427	Buru	M	Adult
AAM0288	FrankfurtSenckenbergNaturalHistoryMuseum	429	Buru	M	Adult
AAM0289	FrankfurtSenckenbergNaturalHistoryMuseum	430	Buru	M	Adult
AAM0294	FrankfurtSenckenbergNaturalHistoryMuseum	7100	Buru	M	Adult
AAM0297	FrankfurtSenckenbergNaturalHistoryMuseum	35645	Buru_or_Sula	M	Adult
AAM0222	DresdenMuseum	3070	Sulawesi	M	Adult
AAM0232	Edinburgh-NationalMuseumsScotland	NMS.Z.1878.3.1	Sulawesi	M	Adult
AAM0234	Edinburgh-NationalMuseumsScotland	NMS.Z.1930.172	North-east Sulawesi	M	Adult
AAM0308	Goteborg_Museum	4.728	North-east Sulawesi	M	Adult
AAM0323	Goteborg_Museum	17.944	North-east Sulawesi	M	Adult
AAM0339	Goteborg_Museum	17.960	North-east Sulawesi	M	Adult
AAM0310	Goteborg_Museum	4.730	North-east Sulawesi	M	Juvenile
AAM0316	Goteborg_Museum	17.937	North-east Sulawesi	M	Sub-adult
AAM0345	Goteborg_Museum	17.966	North-east Sulawesi	F	Adult
AAM0352	Goteborg_Museum	17.973	North-east Sulawesi	F	Sub-adult

**Figure 3.**
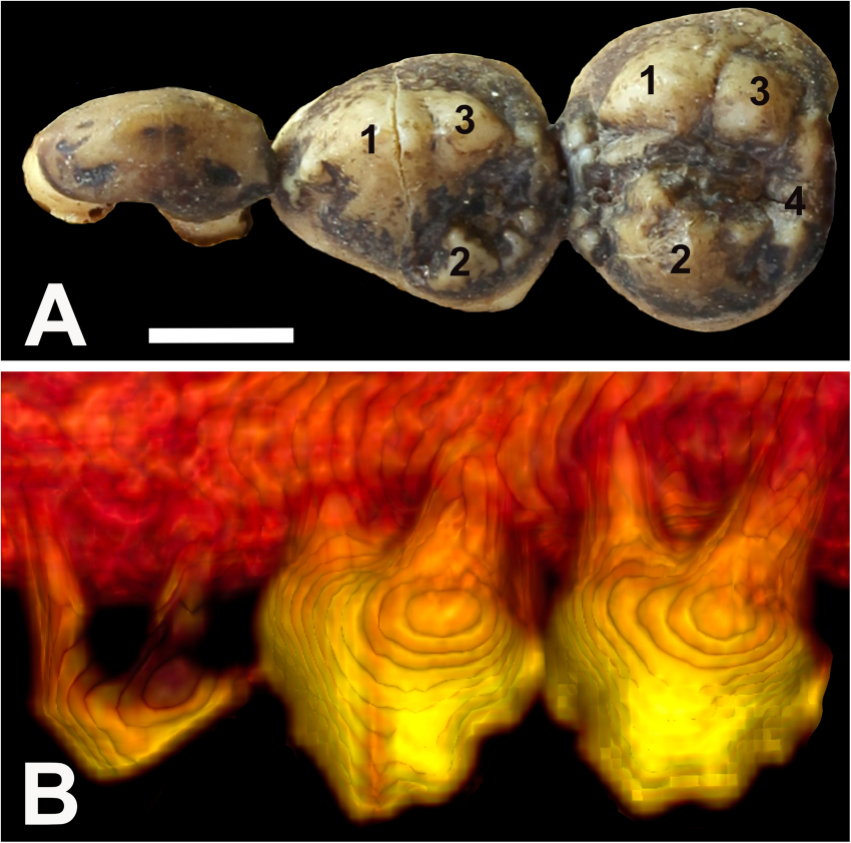
(A) Occlusal view of the left maxillary premolar teeth of Sulawesi babirusa (*Babyrousa celebensis*) (GNM 17.973): persistent deciduous tooth (206), permanent third premolar (207), and permanent fourth premolar (208) (left to right). The list of symbols,^20^ is the number of the cusps of the maxillary premolar teeth—1: paracone; 2: protocone; 3: metacone; 4: tetracone. (B) Three-dimensional lateral CT view of these teeth (bar = 5 mm). Abbreviation: CT, computed tomography.

**Figure 4.**
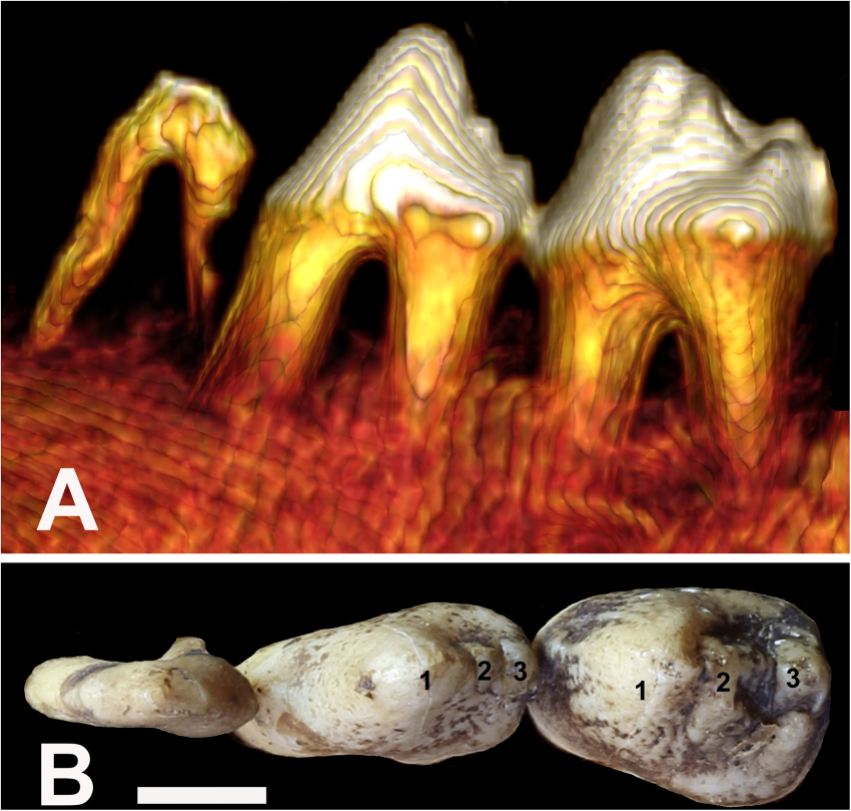
(A) Three-dimensional lateral CT view of the left mandibular premolar teeth of Sulawesi babirusa (*Babyrousa celebensis*) (GNM 17.973): persistent deciduous tooth (306), permanent third premolar (307) and permanent fourth premolar (308) (left to right). (B) Occlusal view of the same left mandibular teeth. The list of symbols,^20^ is the number of the cusps of the mandibular premolar teeth—1: protoconid; 2: metaconid; 3: hypoconid (bar = 5 mm). Abbreviation: CT, computed tomography.

All specimens were photographed, including close-up views of the occlusal surfaces of the teeth. To enable anatomical clarity in the description of observations, the dental anatomical nomenclature of the babirusa teeth was derived from that published for Suidae.^20^ The main cusps on the maxillary premolars are called paracone, protocone, metacone, and tetracone (Figure 3). The main cusps on the mandibular premolars are called protoconid, metaconid, and hypoconid (Figure 4).

For geographical proximity reasons the skulls were CT scanned at 4 different institutions using 4 different CT scanner models.^e–h^

**Figure 5.**
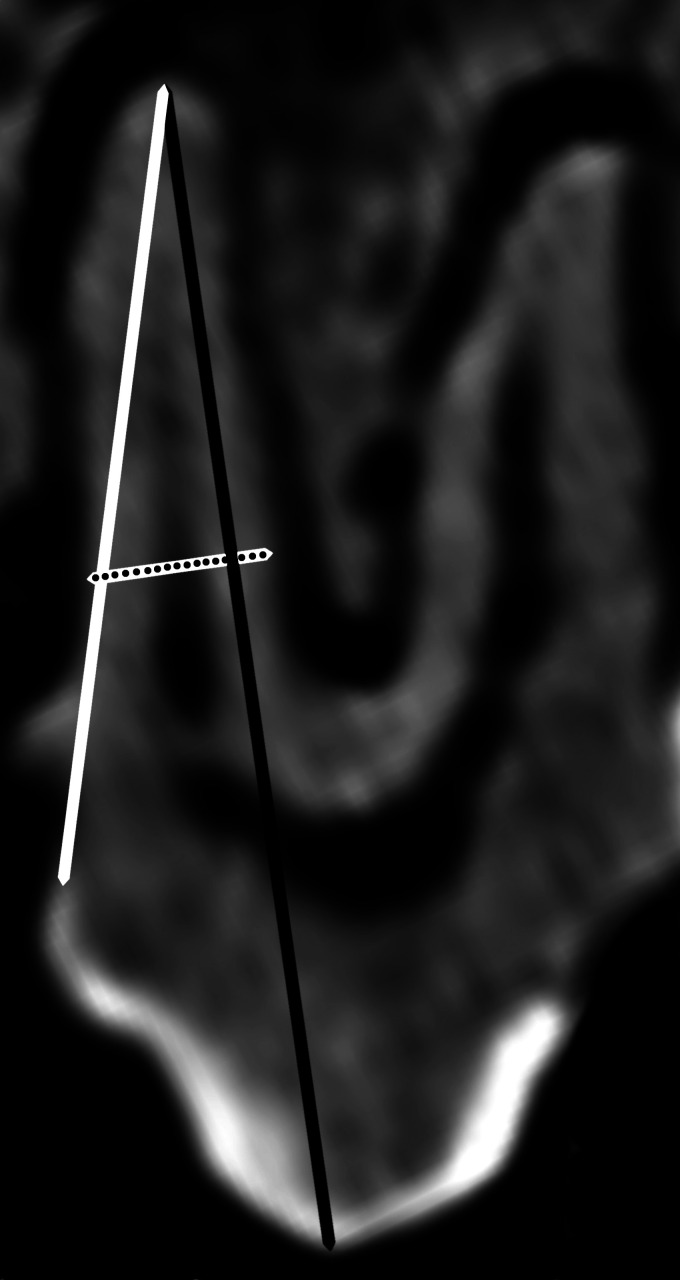
Lateral CT scan of left maxillary tooth (206) of Buru or Sula Island babirusa (*Babyrousa babyrussa*) (NMS,Z,1991.15.3) to illustrate the measurements of tooth length (black), root length (white) and root thickness (dotted). (Size adjusted to column width).

Tooth length was measured from the highest point of the crown (the coronal end) to the apex (the extremity) of the tooth root (Figure 5). Tooth root length was measured from either the buccal or the lingual/ palatal cementoenamel junction (depending on root orientation) to the apex of the root (Figure 5). The occlusal edges of the premolar tooth were formed from the enamel cusps. The occlusal surface “mesial to distal” length and “buccal to lingual or palatal” width of each premolar tooth was measured. Tooth root diameter was measured across the mid length of the tooth root (Figure 5).

Statistical analyses^i^ were undertaken on the teeth from the adult babirusa.

## Results

### Occlusal Morphology

**Figure 6.**
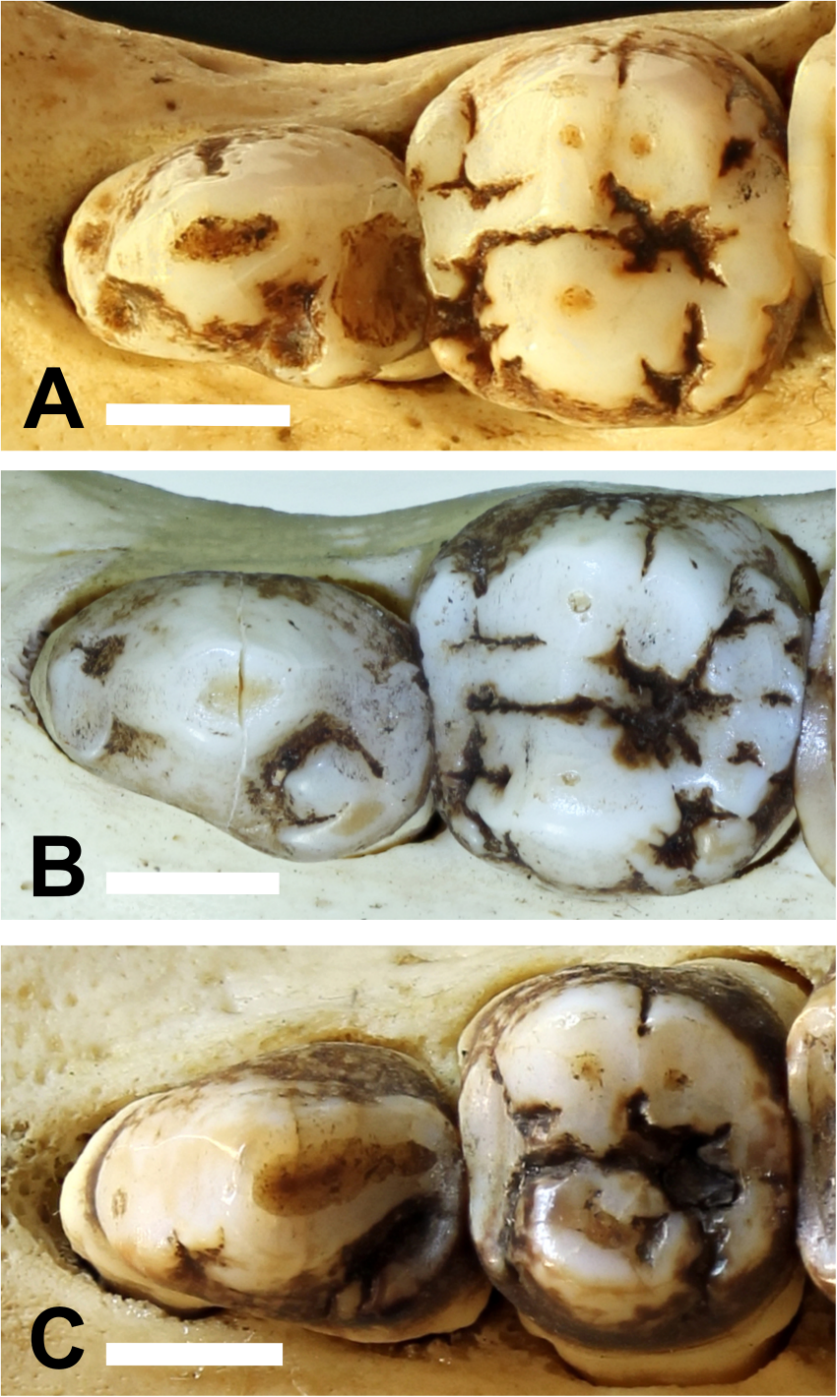
The occlusal surfaces of the left maxillary premolar teeth. (A) Buru or Sula Island babirusa (*Babyrousa babyrussa*) (NMS,Z,1991.15.3). (B) Buru or Sula Island babirusa (*B. babyrussa*) (SMF 35645). (C) Sulawesi babirusa (*Babyrousa celebensis*) (GNM 4.728) (bar = 5 mm).

The occlusal morphology of the permanent maxillary premolar teeth of *B. babyrussa* (Figure 6A and B) was very similar to that of *B. celebensis* (Figure 6C). The enameled crowns of *B. babyrussa* maxillary third premolar teeth (107/207) (n = 34) were longer (9.6 ± 0.7 mm) than they were wide (6.5 ± 0.8 mm) (*P *< .001). Comparable results were found in *B. celebensis* 107/207 (n = 11) (length = 11.1 ± 0.7 mm; width = 7.8 ± 0.7 mm; n = 11; *P *< .001). The enameled crowns of the maxillary fourth premolar teeth (108/208) were rounded (Figure 6); *B. babyrussa* measured 9.9 ± 0.7 mm in length and 9.7 ± 0.7 mm in width (n = 34) (NS). In *B. celebensis* these teeth measured 10.0 ± 1.3 mm in length and 10.1 ± 1.1 mm in width (n = 12) (NS).

The paracone of 107/207 is in the form of a centrally situated, somewhat laterally compressed cone (Figures 3 and 6). On its distobuccal surface lies the slightly smaller metacone (Figure 3). The protocone is smaller still and is situated on the palatal side of the crown (Figures 3 and 6). The paracone 108/208 is bordered distally on the buccal side by a slightly smaller metacone (Figures 3 and 6). The palatal side of the crown is largely occupied by the protocone. Distal to these 3 cusps lies the smaller tetracone. The valleys, or fossids, running distally between the buccal and palatal cusps of 107/207 and 108/208 appear to be a significant feature of these tooth crowns (Figure 3).

**Figure 7.**
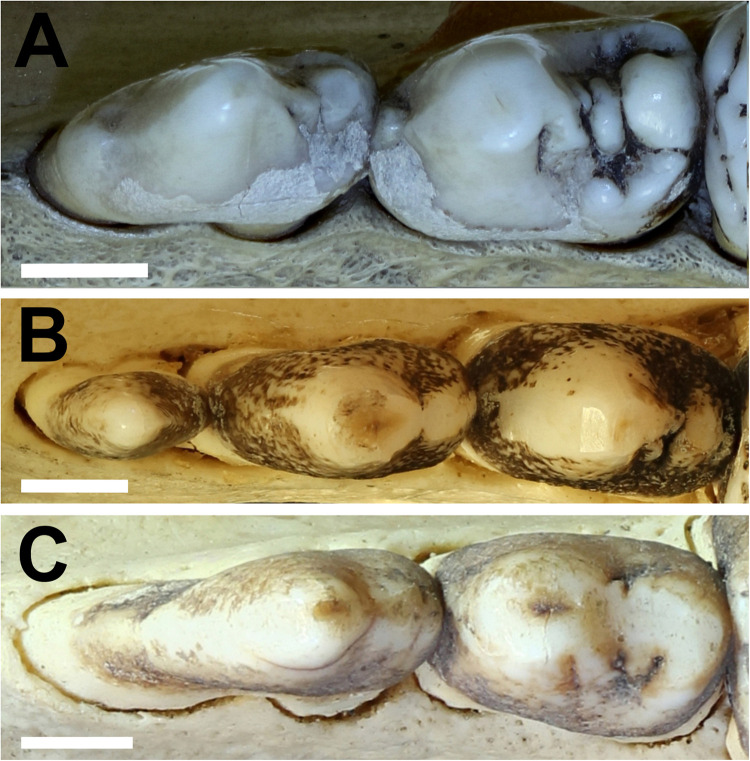
The occlusal surfaces of the left mandibular premolar teeth. (A) Buru or Sula Island babirusa (*Babyrousa babyrussa*) (SMF 7100). (B) Buru or Sula Island babirusa (*B. babyrussa*) (NMS,Z,2002.210.1). (C) Sulawesi babirusa (*Babyrousa celebensis*) (GNM 17.944) (bar = 5 mm).

The occlusal morphology of the permanent mandibular premolar teeth of *B. babyrousa* (Figure 7A and B) is very similar to that of *B. celebensis* (Figure 7C). The enameled crowns of *B. babyrussa* mandibular third premolar teeth (307/407) were longer (10.5 ± 1.0 mm) than they were wide (5.7 ± 0.9 mm) (n = 35; *P *< .001). Comparable results were found in *B. celebensis* 307/407 which were also longer (11.7 ± 1.0 mm) than they were wide (6.1 ± 1.5 mm) (n = 14; *P *< .001). The enameled crowns of the mandibular fourth premolar teeth (308/408) were somewhat elongated; *B. babyrussa* measured 11.9 ± 0.8 mm in length and 7.9 ± 0.8 mm in width (n = 35; *P *< .001). The enameled crowns of 308/408 in *B. celebensis* measured 12.1 ± 1.1 mm in length and 8.1 ± 1.6 mm in width (n = 14; *P *< .001).

The protoconid of 307/407 is the predominant, conical cusp with the small metaconid on its distal slope and the slightly larger hypoconid on the distal edge of the tooth (Figure 4). Together they form a serrated edge to the distal half of the tooth. The protoconid of 308/408 is also the predominant cusp on that tooth, mesially located and occupying about half the occlusal surface area. The metaconid occupies the distal slope of the 308/408 protoconid. The distal edge of the crown supports the small protocone lingually and the tetracone distally.

### Tooth Root Structure

**Figure 8.**
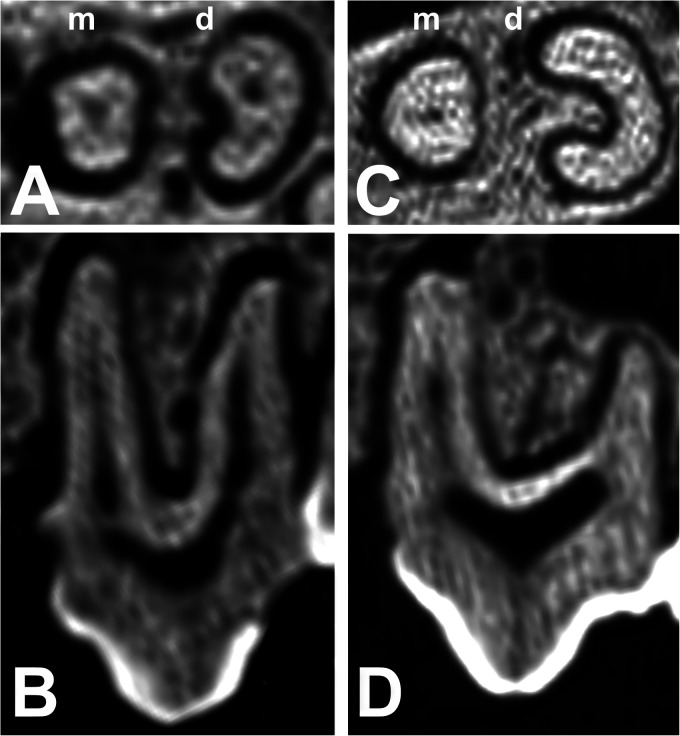
Illustrated differences in the left maxillary third premolar tooth root structure. (A) Cross-sectional CT scan and (B) lateral CT scan of Buru or Sula Island babirusa (*Babyrousa babyrussa*) (NMS,Z,1991.15.3). (C) Cross-sectional CT scan and (D) lateral CT scan of Sulawesi babirusa (*Babyrousa celebensis*) (NMS,Z,1878.3.1) (sizes adjusted to column width). Abbreviations: CT, computed tomography; d, distal; m, mesial.

**Figure 9.**
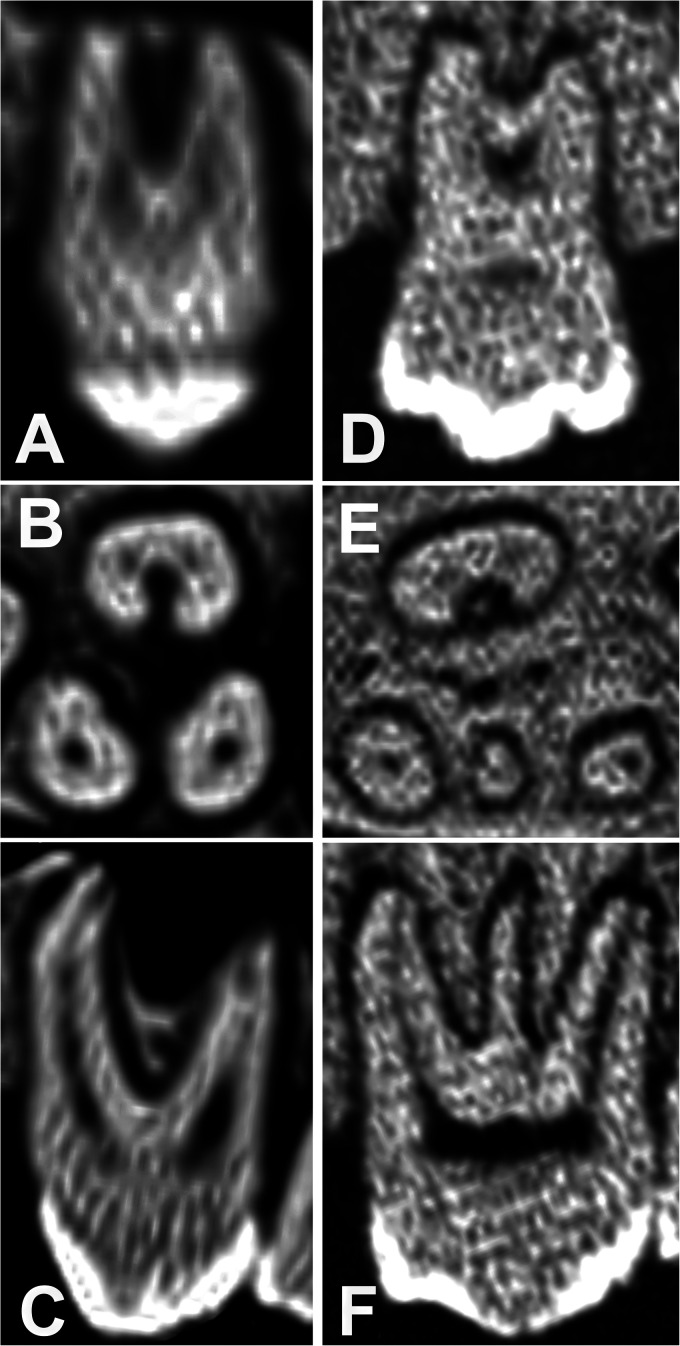
Illustrated differences in the left maxillary fourth premolar tooth root structure. (A) Lateral longitudinal CT scan of the palatal root, (B) cross-section CT of all 3 roots, and (C) lateral CT scan of mesiobuccal and distobuccal roots with 3 roots of Sulawesi babirusa (*Babyrousa celebensis*) (GNM 4.728.). (D) Lateral longitudinal CT scan of the palatal root, (E) cross-section of all 4 roots, and (F) lateral CT scan of the mesiobuccal, centrobuccal, and distobuccal roots of with 4 roots (Buru or Sula Island babirusa [*Babyrousa babyrussa*] [NMS,Z,1992.10.28] [sizes adjusted to column width]). Abbreviation: CT, computed tomography.

The structures of the maxillary premolar tooth roots were either conical, with a variable amount of tapering from the crown to the apex, or alternatively, they were to some degree “C” shaped for a variable proportion of their course from the crown to the apex (Figures 1 and 8). All of the mesial tooth roots of 107/207 and 108/208 were conical, as were all 13 centrobuccal and all distobuccal tooth roots of 108/208 (Figures 8 and 9); each contained a single pulp canal. Often a shallow longitudinal channel ran down the opposing surfaces of the roots (Figure 8A and C). In all but 2 of the 56 distal roots of 107/207 this furrow was sufficiently large to make the root “C” shaped and contain 2 pulp canals. Of the latter, 32 roots tapered obliquely to a single apex, 20 divided into 2 conical structures which tapered to separate apices; the remaining 2 teeth were absent from the skull. The 50 108/208 palatal roots were “C” shaped and contained 2 pulp canals (Figure 9B and E); in 48 of these the roots tapered obliquely to a single apex, while in the other 2 the roots tapered to separate apices (Figure 9).

**Figure 10.**
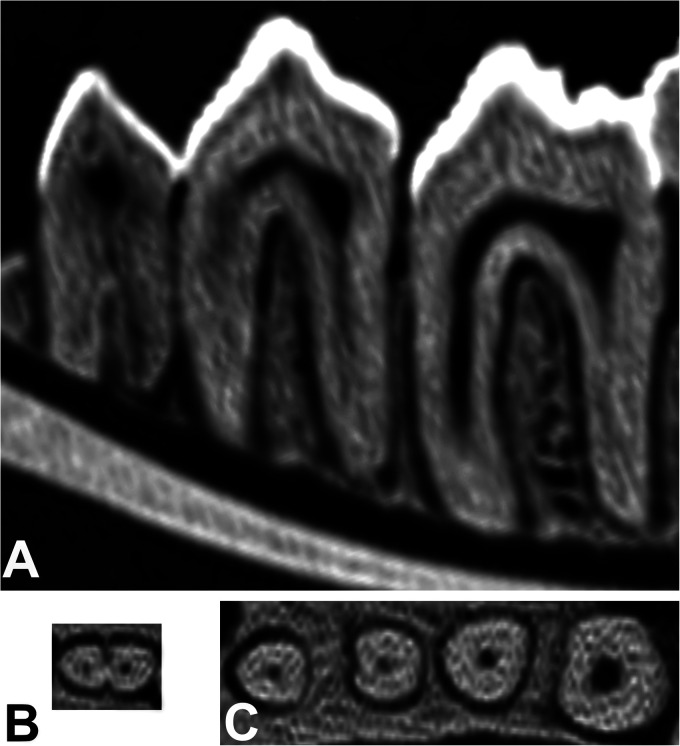
(A) Lateral longitudinal CT scan of the mesial and distal roots of left mandibular persistent deciduous tooth (306), permanent third premolar (307), and permanent fourth premolar (308) (left to right) of Buru or Sula Island babirusa (*Babyrousa babyrussa*) (NMS,Z,2002.210.1). (B) Cross-sectional CT scan of the roots of 306. (C) Cross-sectional CT scan of the roots of 307 and 308 (sizes adjusted to column width). Abbreviation: CT, computed tomography.

The structure of the mesial and distal roots of 307/407 was uniformly conical (Figures 4 and 10). The mesial roots of 308/408 teeth were also conical (Figure 9). However, the distal roots of 308/408 teeth were to some degree longitudinally furrowed or “C” shaped for a variable proportion of their course from the crown to the apex. Two *B. babyrussa* and 4 *B. celebensis* showed 2 pulp canals in the distal root of 308/408.

### Maxillary Tooth Roots, B. babyrussa

Of the 18 skulls, 17 of the 107/207 (94%) had 2 roots (Figure 8); while 1 (6%) had 3 roots. Of the 18 skulls, 12 of the 207 and 14 of the 107 (72%) had 3 roots; 6 of the 207 and 3 of the 107 (25%) had 4 roots (Figure 9); there was no evidence for 1 107 in 1 specimen from Buru (SMF 430). Measurements of the lengths of the teeth, the crown heights, the root lengths, the thickness of the roots, and the diameters of the root apices are summarized in Table 2. The length of 107/207 through the mesial root was longer than through the distal root (*P *< .001), which corresponded to the relative lengths of these 2 roots (*P *< .05). The crown length above the mesial root of the 107/207, represented in part by the paraconal cusp, was longer (*P *< .05) than the crown length above the distal root, represented in part by the metacone. The distal root was thicker than the mesial root (*P *< .001) but narrowed to a smaller apex (*P *< .05). The length of 108/208 through the mesiobuccal root was also longer than through the distobuccal and palatal roots (*P *< .001). The crown height of the mesiobuccal root, represented in part by the paraconal cusp, was longer (*P *< .001) than the crown length above the palatal root, represented in part by the protocone. There was no difference in either length or root thickness between the 3 roots of 108/208. The palatal root apices were larger than those of the mesiobuccal (*P *< .001) and distobuccal (*P *< .05) roots.

**Table 2.** Adult Babirusa (*Babyrousa babyrussa* and *Babyrousa celebensis*) Maxillary Premolar Teeth Measurements.

Maxillary tooth ID	107/207	107/207	108/208	108/208	108/208
Maxillary tooth roots	Mesial	Distal	Mesiobuccal	Distobuccal	Distolingual
*B. babyrussa* (male)					
Tooth length—Av ± Std mm (n)	16.2 ± 2.0 (31)***	14.9 ± 1.8 (31)***	16.7 ± 1.5 (34)***	14.1 ± 1.4 (32)***	14.7 ± 1.1 (33)***
Crown height—Av ± Std mm (n)	4.5 ± 1.5 (30)*	3.7 ± 1.4 (30)*	4.9 ± 1.6 (34)***	2.3 ± 1.3 (32)***	3.7 ± 1.1 (33)***
Tooth root lengths—Av ± Std mm (n)	11.8 ± 1.5 (31)*	11.2 ± 1.0 (32)*	11.8 ± 0.7 (34)	11.8 ± 1.6 (32)	11.0 ± 0.9 (33)
Tooth root thickness—Av ± Std mm (n)	4.3 ± 0.4 (31)***	5.5 ± 0.5 (31)***	3.7 ± 0.6 (34)	3.6 ± 0.4 (34)	3.8 ± 0.8 (34)
Tooth root apex diameter—Av ± Std mm (n)	2.1 ± 0.4 (31)*	1.8 ± 0.7 (31)*	1.6 ± 0.3 (34)***	1.7 ± 0.4 (34)	1.9 ± 0.5 (33)***
*B. celebensis* (male)					
Tooth length—Av ± Std mm (n)	16.0 ± 2.0 (11)	15.4 ± 2.7 (11)	18.0 ± 1.2 (11)***	16.2 ± 1.6 (8)	14.8 ± 1.1 (9)***
Crown height—Av ± Std mm (n)	5.3 ± 0.9 (9)	4.8 ± 2.0 (10)	6.0 ± 1.5 (10**	4.2 ± 1.4 (7)	3.9 ± 1.1 (8)**
Tooth root lengths—Av ± Std mm (n)	11.2 ± 1.2 (11)	10.7 ± 1.2 (11)	11.9 ± 1.3 (11)	11.3 ± 1.7 (10)	10.4 ± 2.1 (10)
Tooth root thickness—Av ± Std mm (n)	4.5 ± 0.4 (11)*	5.7 ± 1.5 (11)*	4.2 ± 0.5 (11)***	3.4 ± 0.5 (11)***	3.4 ± 0.5 (11)***
Tooth root apex diameter—Av ± Std mm (n)	2.2 ± 0.8 (11)**	1.4 ± 0.1 (9)**	1.7 ± 0.4 (11)	1.5 ± 0.4 (10)	1.4 ± 0.4 (11)

* *P* < .05; ** *P* < .01; *** *P* < .001.

Abbreviations: Av, average; Std, standard.

### Maxillary Tooth Roots, B. celebensis

In all 14 skulls, 107 and 207 had 2 roots. Ten of the 108 and 208 (71%) had 3 roots. One bilateral pair of 108/208 (7%) had 4 roots. One bilateral pair of teeth (7%) was unclear. Measurements of the lengths of the teeth, the crown heights, the root lengths, the thickness of the roots, and the diameters of the root apices are summarized in Table 2. No differences between roots in either tooth length or tooth root length were detected in 107/207. There was also no difference in crown heights. However, the distal root was thicker than the mesial root (*P* < .05) but narrowed to a smaller apex (*P *< .001). The lengths of 108/208 through the mesiobuccal (*P *< .001) and the distobuccal (*P *< .01) were longer than through the palatal root. The crown height of the mesiobuccal root, represented in part by the paraconal cusp, was longer (*P *< .01) than the crown height above the palatal root, represented in part by the protocone (Table 2). The distal roots of 107/207 were thicker than the mesial roots (*P *< .05) but narrowed to a smaller apex (*P *< .01). The mesial roots of 108/208 were thicker than those of the distobuccal (*P *< .001) and palatal (*P *< .001) roots. No difference in the sizes of their tooth root apices was detected. The subadult babirusa 107 and 207 had 2 roots, while the 108 and 208 all had 3 roots.

### Mandibular Tooth Roots, B. babyrussa

Of the 18 mandibles, 17 of the 307 had 2 roots, 1 had 3 roots, and all 407 had 2 roots. In all 18 mandibles, 308 and 408 had 2 roots (Figure 10). Measurements of the lengths of the teeth, the crown heights, the root lengths, the thickness of the roots, and the diameters of the root apices are summarized in Table 3. The lengths of 307/407 through the distal roots were longer (*P *< .001) than through the mesial roots. The crown height above the distal root, represented in part by the metacone, was longer (*P *< .01) than the crown height over the mesial root of 307/407, represented in part by the paraconal cusp. However, the lengths of 308/408 through the mesial roots were longer (*P *< .001) than through the distal roots and the crown height over the mesial root was also longer than over the distal root (*P *< .01). There were no differences in length between the roots of 307/407 and 308/408 (Table 3). The thicknesses of the distal roots and root apices of both teeth were greater (*P *< .001) than for the corresponding mesial roots.

**Table 3.** Adult Babirusa (*Babyrousa babyrussa* and *Babyrousa celebensis*) Mandibular Premolar Teeth Measurements.

Mandibular tooth ID	307/407	307/407	308/408	308/408
Mandibular tooth roots	Mesial	Distal	Mesial	Distal
*B. babyrussa*				
Tooth lengths—Av ± Std mm (n)	19.7 ± 2.5 (32)***	21.1 ± 2.0 (36)***	22.8 ± 1.8 (36)***	19.5 ± 2.4 (36)***
Crown height—Av ± Std mm (n)	4.3 ± 1.4 (30)**	5.5 ± 1.9 (35)**	6.0 ± 1.9 (36)**	5.1 ± 1.8 (36)**
Tooth root lengths—Av ± Std mm (n)	15.7 ± 1.9 (32)	15.7 ± 1.7 (36)	16.8 ± 1.3 (36)***	14.4 ± 1.2 (36)***
Tooth root diameter—Av ± Std mm (n)	3.2 ± 0.5 (32)***	4.1 ± 0.5 (36)***	4.3 ± 0.7 (36)***	5.4 ± 0.8 (36)***
Tooth root apex diameter—Av ± Std mm (n)	1.5 ± 0.5 (36)***	2.5 ± 0.6 (36)***	1.7 ± 0.5 (36)***	3.5 ± 0.8 (36)***
*B. celebensis*				
Tooth lengths—Av ± Std mm (n)	19.0 ± 2.0 (11)***	21.0 ± 0.9 (13)***	22.3 ± 2.2 (13)*	20.0 ± 2.1 (13)*
Crown height—Av ± Std mm (n)	4.7 ± 1.7 (10)***	7.8 ± 1.1 (12)***	5.7 ± 1.3 (12)	6.1 ± 2.5 (12)
Tooth root lengths—Av ± Std mm (n)	14.1 ± 1.2 (13)**	13.2 ± 1.1 (13)**	16.6 ± 1.4 (13)***	14.0 ± 1.1 (13)***
Tooth root diameter—Av ± Std mm (n)	3.5 ± 0.4 (13)***	4.8 ± 0.5 (13)***	5.0 ± 0.9 (13)**	5.7 ± 1.1 (13)**
Tooth root apex diameter—Av ± Std mm (n)	2.3 ± 0.7 (13)***	3.5 ± 0.8 (13)***	2.4 ± 0.7 (13)**	3.1 ± 0.6 (13)**

* *P* < .05; ** *P* < .01; *** *P* < .001.

Abbreviations: Av, average; Std, standard.

### Mandibular Tooth Roots, B. celebensis

In all 14 skulls, 307 and 407 had 2 roots. Likewise, in all of the 14 skulls 308 and 408 had 2 roots. Measurements of the lengths of the teeth, the crown heights, the root lengths, the thickness of the roots, and the diameters of the root apices are summarized in Table 3. The lengths of 307/407 through the distal roots were longer (*P *< .001) than through the mesial roots, and the crown heights above the distal roots were also longer than those over the mesial roots (*P *< .001). The lengths of 308/408 through the mesial roots were longer (*P *< .05) than through the distal roots. The mesial roots of 307/407 and 308/408 were longer (*P *< .01 and *P *< .001, respectively) than the distal roots. The thicknesses of the distal roots and root apices of both teeth were greater (*P *< .001 and *P *< .01, respectively) than for the corresponding mesial roots. The juvenile and subadult babirusa had 2 roots for the 307/407 and 308/408.

### Pulp Chambers

**Figure 11.**
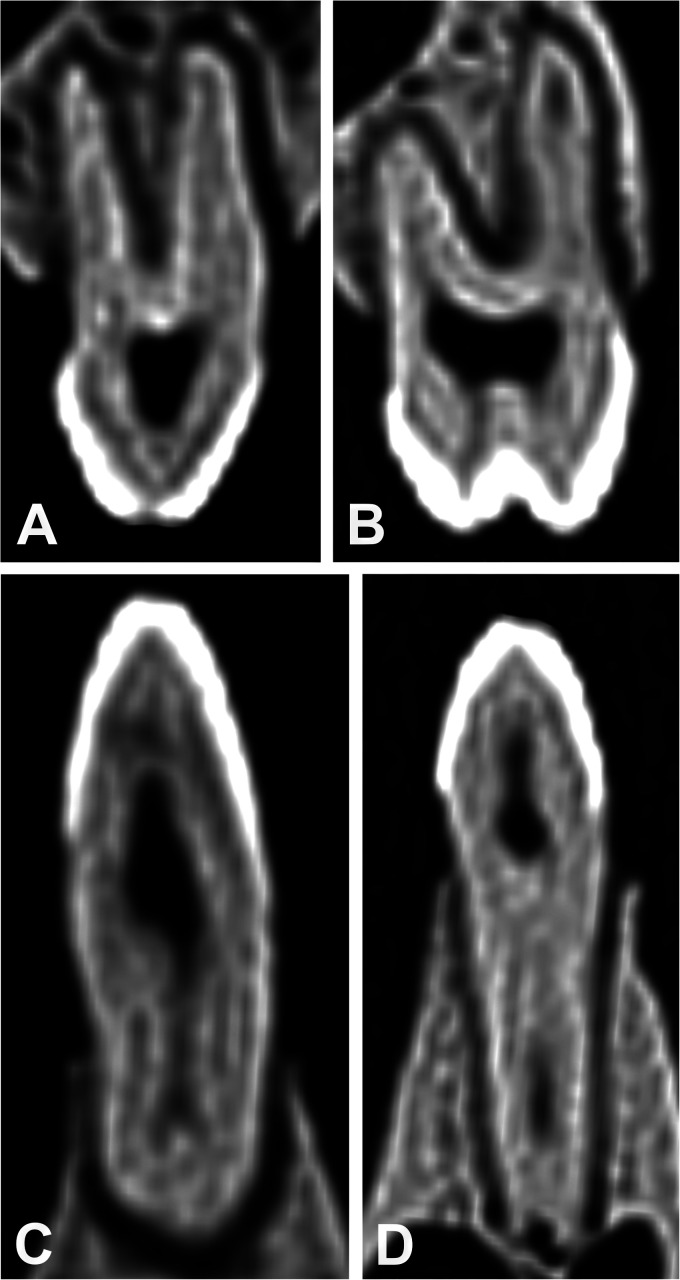
Mesial longitudinal CT scans of the left premolar teeth illustrating the (opaque) pulp chambers below the enamel and dentine of the cusps. (A) 207 of Sulawesi babirusa (*Babyrousa celebensis*) (GNM 4.728) illustrating the apparent split in the mesial root caused by a fold down its distal surface. (B) 208 of Buru or Sula Island babirusa (*Babyrousa babyrussa*) (NMS,Z,2002.210.1) illustrating the mesiobuccal (left) and palatal (right) roots. (C) 307 of Sulawesi babirusa (*B. celebensis*) (NMS,Z,1878.3.1) illustrating the ridged roof of the pulp chamber. (D) 308 of Buru or Sula Island babirusa (*B. babyrussa*) (NMS,Z,2002.210.1) illustrating the penetration of the pulp chamber under the enamel and dentine of the cusps.

The pulp chambers of 107/207 were arched and elongated regions positioned under the paracone and metacone (Figure 3). The ridged roof of the chambers lay above the junction of the crown and the root of the tooth (Figures 8B, 8D, and 11A). The floors of the pulp chambers were served by the rod-shaped mesial roots via the funnel end of a single, approximately axially located pulp canal (Figure 8). From the “C-shaped” distal roots the pulp chambers were supplied (in all but 1 skull) by 2 pulp canals.

The pulp chambers of 108/208 teeth coalesced under the 3 main cusps of the tooth, the paracone, protocone, and metacone (Figure 3). The roof of the chamber was irregular in shape and was situated at or above the junction of the crown and the root of the tooth (Figures 8F and 11B). The pulp chamber formed a wide and deep space into which fed the funnel-ended pulp canals from the 3 or more tooth roots (Figure 9). Each of the “C-shaped” palatal roots contained 2 pulp canals. Each of the 13 medial/accessory roots contained 1 pulp canal.

The elongated pulp chamber of 307/407 tooth was largely situated under the protoconid (Figure 4). The ridged roof of the chamber extended up under the cusp and its floor lay below the junction of the crown and the root of the tooth (Figures 10 and 11C). In all *B. babyrussa* teeth there was a single centrally located pulp canal in each of the mesial and distal roots. In 2 *B. celebensis* skulls the distal tooth roots bilaterally contained 2 pulp canals.

The pulp chamber of 308/408 tooth broadened mesially and distally under the protoconid and metaconid (Figure 4). In all teeth most of the volume of the chamber was situated below the junction of the crown and the root of the tooth (Figure 10A), but its roof could extend up into the dentine under the cusps (Figure 11D). The chamber was uniformly supplied from the mesial tooth root by 1 centrally located pulp canal; in all but 3 of the 36 of the distal roots of *B. babyrussa* teeth and in 7 of the 14 distal roots of *B. celebensis* teeth there was a single pulp canal; in the other 7 teeth there were 2 pulp canals. Each of the 3 medial roots contained 1 pulp canal.

## Discussion

This study is the first since those from 1900^8,9^ to have closely examined the structure of the maxillary and mandibular premolar teeth in the adult babirusa (*Babyrousa* spp*.*). It has extended, by illustration and measurement, previously observed descriptions of these teeth^8,9^ and has provided additional detail regarding the anatomical variation of their roots. It has confirmed that for both *Babyrousa* spp. the roots of these teeth were more complex than simple conical structures (Figures 8 and 9). Whereas the mesial root of the mesiodistally elongated 107/207 did have a conical type of structure, the distal root was most often “C” shaped. The roots of the 108/208 tooth also included a “C”-shaped component, on its palatal side. By way of contrast, the mesial and distal roots of the mandibular premolar teeth were all tapering, conical structures, deeply anchored in the bone (Table 3 and Figure 10). This corresponded to the measurements reported for domestic pig *Sus domesticus*^21,22^ and to the results of the radiographic study on the Large White breed of pig.^23^

It has long been recognized that the babirusa has fewer premolar teeth (2 in the maxilla and 2 in the mandible on each side; premolars 1 and 2 are missing) than *Potamochoerus* spp.,^24^ which have 3 or 4 in the maxilla and 2 or 3 in the mandible, and *Sus scrofa* which has 4 upper and 4 lower premolars on each side.^7,8^ Therefore, it was of interest to know how these were rooted. One study reported that the maxillary third and fourth premolars of the Chinese experimental miniature pig (*Sus domesticus*) have 3 to 4 roots.^25^ An earlier and more detailed study, published that the domestic pig maxillary first premolar tooth had 2 roots, the second and third premolars had 3 each, and that the fourth premolar teeth had 4 roots.^22^ The maxillary third premolar teeth of the Japanese wild pig (*Sus scrofa leucomystax)*^26^ had 3 roots, 1 mesial, 1 distobuccal, and 1 distopalatal, and the fourth premolar teeth had 4 roots, 2 on the buccal side and 2 on the palatal side.^27^ The maxillary third premolar teeth of *Sus* corresponds to teeth 107 and 207 of *Babyrousa* (Figure 1). Thus, with 33 observations of 3 roots in the maxillary third premolar teeth of *Sus*,^22^ there was consistently 1 root more in this tooth of *S. domesticus*^21^ than the 2 roots found in all but one of the equivalent *Babyrousa* teeth. The maxillary fourth premolar teeth of *Sus* correspond to teeth 108 and 208 of *Babyrousa* (Figure 2). These maxillary fourth premolar teeth have been reported to have 3 (5 times = 15%), 4 (21 times = 64%), 5 (5 times = 15%), and 6 roots (twice = 6%) (Table 1).^22^ Our equivalent observations for *B. babyrussa* were that 26 108/208 (74%) had 3 roots and 9 (25%) had 4 roots, and for *B. celebensis*, 10 teeth (83%) had 3 roots and 2 teeth (16%) had 4 roots. In another study, it was reported that the maxilla of wild *S. scrofa* had 2 roots in the first premolar, 3 roots in the second and third premolars, and 3 roots in the fourth premolars, of which 2 roots were usually forked so that a total of 5 root tips occurred.^28^ In unpublished studies on maxillae of 2 adult male *Sus celebensis* the authors found that the maxillary third premolars had 2 roots and the fourth maxillary premolars had 3 or 4 roots.

A previous study reported that in the mandible of domestic pigs the first premolar had 1 or 2 roots, the second premolar had 2 roots, and the third premolar was found to have 2 (20 times = 69%) and 3 roots (9 times = 31%), the fourth premolar always had 3 roots (29 times).^22^ In contrast, a separate study indicated that in mandible of the “Clawn” strain of miniature Japanese domestic pig, the second, third, and fourth premolar teeth had 2 roots.^29^ The same observations were also made for the second and third premolar teeth in the Japanese wild pig, but 3 roots were found in the fourth premolar teeth.^27^ For the mandible of the European wild pig, *S. scrofa*, 2 roots for the first and second premolar teeth, and 3 roots for the third and fourth premolar teeth have been reported.^30^ The third mandibular premolar teeth of *Sus* corresponded to 307 and 407, and the fourth premolar teeth corresponded to 308 and 408 in *Babyrousa*. This study found that 35 of the 307 and 407 teeth in *B. babyrussa* had 2 roots and 1 had 3 roots, whereas in *B. celebensis* all of the mandibular premolar teeth had 2 roots. In unpublished studies on the mandibles of 2 adult male *S. celebensis* the authors found that both the third and fourth premolar teeth had 2 roots.

The pulp canals described in the current study lay within the scale of resolution of the CT scans used. A single well-defined canal was seen running largely axially from the root apex to the pulp chamber in all mesial roots of the first premolar teeth (Figure 8). A similar finding was made in a micro-CT study of the mandibular teeth of miniature *S. domesticus*.^29^ It also reported that the distal root of the fourth mandibular premolar teeth had 2 root canals. Thin and indistinctly “ghosted” opacities were seen to be associated with a number of the palatal roots of 108/208 in the present study. These raised the hypothesis of further intra-root pulp-canal complexity. The higher resolution offered by micro-CT studies has revealed that individual human premolar maxillary tooth roots do show considerable variability in the number, size, shape, pattern, and interconnectivity of pulp canals contained within them; the root canal system is not a single canal running uniformly from pulp chamber to apex.^31–33^ Methods have subsequently been devised to describe this complex variation within human premolar teeth.^34–36^ These methods offer alternative frameworks for comparable descriptions and further veterinary analyses of the maxillary teeth of babirusa and other wild pig species in the future.

The cutting function of the premolar teeth of *S. scrofa* has been long commented upon.^8,22^ Although the equivalent teeth of the babirusa were morphologically more rounded, the relatively narrow longitudinal shape of the maxillary third premolar tooth and both opposing mandibular premolar teeth supports this view (Figures 3 and 4). The fossids (Figure 3), running distally between the buccal and palatal cusps of 107/207 and 108/208, and the somewhat serrated arrangement of distal cusps of maxillary and mandibular premolar teeth (Figures 3 and 4) also contribute to this conclusion. Studies of the feeding behavior of babirusa in zoological collections^37,38^ and in the wild^3,39^ have placed emphasis on leaf and fruit browsing behavior. *B*. *celebensis* in zoological collections have been observed cropping the leaves off bramble bushes (*Rubus* sp. L.) and low-hanging cherry trees (*Prunus* sp. L.),^38^ and have been seen standing on their hind limbs to browse on the leaves of taller trees^40^ and swimming in water courses to access freshwater lettuce (*Pistia stratiotes* L.).^41^ *B. babyrussa* on Buru have been observed biting soft leaves off low-lying (unidentified) plants and creepers as they walked past them.^12^ Other food substances reported from Buru and the Sula Islands included the leaves of Cyanthea [*Alsophyla*] *glauca* Bory and *Homalomena pendula* (Blume) Bakh.f., the low-hanging fruits of various fig trees (*Ficus* spp. L.), the sweet olot (*Hornstedtia rumphii* Sm.) and the fruit of *Rubus fraxinifolius* (Poiret).^3^ The biting of various types of vegetation for nest-building has also been reported.^42^ The occlusal shapes of the premolar teeth contribute to an ease-of-carrying function, and the depths of the roots indicate inherent strength and stability. The relative lack of wear with age of the occlusal surfaces of the premolar teeth when compared to the molar teeth suggests that the premolar teeth are mainly involved in cutting relatively soft plant material.^12^ In contrast, the elongated chisel-like mandibular incisors are used to cut through tougher plant material, such as sweet potato (*Ipomoea batatas* (L.) Lam.) and coconut (*Cocos nucifera* L.) kernel and haustorium.^41,43^ Tooth wear profiles indicate that the cracking of seeds and nuts as well as the mastication of foods are largely undertaken by the molar teeth.^12,13^ However, the rounded enamel crowns and additional roots of the maxillary third premolar teeth suggest that these teeth may contribute toward the food crushing functions of babirusa molar teeth.

## Conclusions

The relative structure of the mandibular third premolar teeth in babirusa suggest that although a cutting function appears to be present, it may play a sufficient but subsidiary role to that of food grinding and crushing by the maxillary fourth premolar teeth. The occlusal surfaces, tooth root number and the physical structures of the roots of the fourth premolar teeth would appear to support such a hypothesis.
